# The Roles of Financial Threat, Social Support, Work Stress, and Mental Distress in Dairy Farmers’ Expectations of Injury

**DOI:** 10.3389/fpubh.2016.00126

**Published:** 2016-06-21

**Authors:** Emilia M. Furey, Denis O’Hora, John McNamara, Stephen Kinsella, Chris Noone

**Affiliations:** ^1^School of Psychology, National University of Ireland, Galway, Ireland; ^2^Teagasc, Kildalton College, Piltown, Kilkenny, Ireland; ^3^Department of Economics, University of Limerick, Limerick, Ireland

**Keywords:** farming, dairy, injury, mental health, financial threat

## Abstract

Farming is dangerous, with fatalities among the highest in any occupation. Farmers often work alone, for long hours, with unreliable equipment and in difficult weather conditions with hazardous chemicals and livestock. In addition, farmers make large financial commitments exposing them to high levels of financial risk. Exposure to such financial risk can give rise to subjective experiences of financial threat (FT) that are psychologically challenging. The current study attempted to characterize the role that FT plays in farm injuries. One hundred and twenty one dairy farmers completed a battery of questionnaires assessing FT, social support (SS), depression, anxiety, farm job stress, and health and safety beliefs. Mental distress directly predicted farmers’ expectations of injury and a direct effect of non-financial farm stress (FS) approached significance. Mental distress mediated these relationships as evidenced by significant indirect effects of FS and FT, and SS served to reduce distress. These findings support calls for interventions designed to reduce FS and FT and increase SS for farmers.

## Introduction

Farming is one of the most dangerous occupation in Ireland (1) and worldwide (2); with fatalities five times higher than construction, with self-employed or family farmers at significantly increased risk. In Ireland, 30 people were killed in farm-related incidents in 2014, a rise of 87% from the previous year, and these deaths accounted for ~55% of all the work related deaths that year. According to the International Labor Office (2), of ~335,000 workplace fatalities every year, 170,000 (over 50%) are in agriculture. In order to help develop policy and personal interventions reduce farm accidents, the current study sought to assess psychological factors that influence farmers’ expectations of injury. In light of recent market changes, the study focused in particular on the effects of financial threat (FT) on Irish dairy farmers.

Among farmers, dairy farmers are of the groups most at risk from workplace accidents. In Ireland, dairy farmers are over-represented in farming fatalities. According to McNamara (3), 58% of Irish farming fatalities in the period 2000–2007 took place on dairy farms, while dairy farms constitute ~11.2% of farms (4). Dairy farming is conducted on 15,600 farms in Ireland, and the average farm size is 55.9 ha with 76.4% within the 30–99 ha range. In terms of economic activity, dairy farms are 3.75 times larger than the average farm (4). Dairy farms are highly capital intensive deploying on average €0.98 million worth of assets comprised of land and buildings (85.6%), livestock (7.2%), machinery (4.9%), and trading assets [2.3%; (5)]. Despite their economic size, the average labor units deployed on dairy farms is estimated at 1.59 labor units, with 85.5% being farm family, principally the farm operator, who provide an estimated 57% of total labor (6).

Stress (both physical and psychological) is a strong predictor of farm injury and resulting safety behaviors (7), as well being a connector between financial problems and injury in farming (8). A number of features of dairy farming expose these farmers to greater financial instability and potential stress than farmers in other sectors. First, the constant work commitment associated with milking production constitutes a consistent burden (9). Second, dairy farmers have greater exposure to uncontrollable external factors, such as the weather, sick animals, government policy, and the economy (10). Finally, in recent years, dairy farmers have faced high levels of financial instability (11).

For Irish dairy farmers, changes in the financial stability of the milk price have lead to greater price uncertainty for farmers. Since 2008, a series of protections that were in place to protect milk price from lower price competition from outside Europe have been progressively removed leading to increased volatility in the milk price. For the period 1993–2006, French and Shalloo (12) demonstrated that the milk price was relatively stable, at 17% change across the 13-year period, but, in the following period, 2007–2015, there was considerable variability (91%; from 22 to 42 cent per liter) in milk price (13). The removal of the EU milk quota system in 2015 also has had implications for farmers’ financial stability. In 1984, EU policy set a pan-European limit on milk production using a milk quota to limit surplus production (14). Each EU country was allocated a national quota to be divided among dairy farmers. Farmers who exceeded quota in a production year were required to pay a national levy. The effects of the quota on dairy farming in Ireland were manifold and complex (15). However, in April 2015, the EU milk quota system was removed. The removal of the quota has potential positive and negative effects. The dairy quota system was intended to provide price and supply stability by limiting production, but these market-distorting restrictions on production had negative effects, such as dumping of excess milk. The removal of the quota, and associated price supports, therefore, constituted an opportunity for greater earnings through increased production, but also there was concern that greater exposure to world market conditions would lead to significant falls in the price obtained for milk. Thus, the downside of the removal of the quota is potential increased price instability for dairy farmers (i.e., they were less sure how much their milk would sell for) and increased financial uncertainty.

Nearly 25% of farmers report financial problems and nearly 80% are most worried about money (16). Melberg (17) identified the main stressors among farmers as: their evaluation of the state of the household economy, presence of unsafe working conditions, injury, ill–health, or disability. High reported levels of stress and stress symptoms (combined with low engagement with safety behaviors) have been shown to predict potential risk of injury in farmers (18). Farmers (aged 55–60 years old) were found to report high emotional stress and mental ill-health in relation to health and safety needs (19), with a significant relationship found between self-reported stress and injury. In particular, exposure to high levels of financial uncertainty may induce subjective experiences of FT that are psychologically challenging but, to date, there is little research that specifically addresses these issue in the farming community.

The job demands-resources model [JDR; (20)] provides a useful descriptive framework for conceptualizing the effects of workplace stress on farmers. It has been supported in many studies across different job contexts (21). The core principle of the theory is that job demands can incur psychosocial and physical costs, but job resources trigger motivation processes that lead to greater work engagement and performance and that can offset the costs incurred by job demands (22). As self-employed lone workers, farmers have fewer resources, both practical and psychological, to deal with negative workplace situations than the average worker. In terms of demands, farmers are exposed to higher levels of stress and the effects of such stress are observed in negative physical and mental health outcomes. Previous studies relating the JDR model to safety at work found that the effects of job resources and job demands on safety outcomes were mediated by emotional exhaustion, such that greater resources protected against emotional exhaustion, which was associated with better safety outcomes, while higher demands increased emotional exhaustion, which led to more negative safety outcomes (23).

An individual’s mental health constitutes an indicator of the psychological resources that he or she can employ at work. In line with the foregoing theoretical position, reduced mental health should predict injury and a number of studies have, in fact, demonstrated an association between mental health and occupational injury in farming (24) and other industries (25, 26). Male farmers have been found to have higher levels of anxiety and depression compared with matched controls (27, 28). Farmers have higher incidence rates of suicide and psychological distress, and lower use of health services that provide support for those with mental ill-health (29, 30). Depression is associated with injury in farming (31) and those farmers who suffer with depression are more likely to experience injury and less likely to engage in safety behaviors when farming injury (32) leaving them susceptible to injury. Anxiety and depression have been associated with impaired work performance and safety (33).

Traditionally, an important source of resources for the farmer has been the informal networks of farmers and other social supports (SSs) at home and in the local community. Economic, physical, and psychological supports were provided in this way. In relation to mental health, SS has been found as the most important predictor of subjective wellbeing for men in rural communities (34), as SS is beneficial to farmers’ mental health (35). It works as a protective factor, reducing the probability and severity of mental health problems (36). A farmer’s level of SS has been shown to affect the level of risky behaviors (such as operating without safety equipment) the farmer is willing to engage in (32). SS may act as a “buffer” against the negative effects of stress, such as ill-mental health, for middle-aged males (37). Farmers’ mental health seems to be moderately protected by being in a relationship or having someone to consult (38). Finally, spousal support has been found to protect farmers from stress; buffering the effects of economic pressure on the men that reduces depression (39) and protects against the incidence of anxiety (40).

Dairy farmers are exposed to a range of potential stressors. Of particular interest, in the current study, were the effects that financial worries may have on mental health and farmers’ health and safety behaviors. As a measure of health and safety behaviors, we focused on farmers’ expectations of injury. In line with the foregoing theories, we hypothesized that mental health may mediate the effects of FT and FS on susceptibility to injury. Strong mental health may function to reduce the impact and effect of financial worries and other stressors on injury expectations; possibly buffering the effect of stress on behavior by using mental resources to deal with the negative effects of stress (41). Conversely, FT and FS may cause mental distress (i.e., reduced mental health), which reduces the resources available to the farmer to engage in safe behavior and increase injury expectations. SS, on the other hand, increases available resources, decreasing mental distress and injury expectations. To summarize, the current study assessed the negative effects of FS and financial worries and the positive effects of SS on self-reported injury expectations and assessed whether these effects were mediated by mental distress.

## Materials and Methods

### Participants

All participants were active farming men (*n* = 121) ranging in age from 18 to 80. The 45- to 54-year-old group (33.9%, *n* = 41) was the most common age category. Participants’ ethnicity comprised Euro-Caucasian (*n* = 121). Exclusion criteria included female farmers; farmers who rented and did not own farms; farmers not from Ireland (non-national farmers), and if farmers were below 18 years old or above 80 years old. Female participants were excluded as data indicate that the majority of dairy farmers are recognized as male (31) and it would have been difficult to balance the gender ratio. Farmers renting out land do not have the same personal financial liability as farmers who own their land, and so they were excluded. Farmers originally from outside Ireland were excluded too, since they may have had alternative training and knowledge of health and safety in farming from those originating in Ireland. Ages outside of 18–80 cohort may have had additional influences that confounded the effect of the variables on the dependent variable, such as health issues or the effects of old age, on farming abilities and so they were excluded.

Relevant details of participants are provided in Table 1. In summary, the farmers surveyed constituted a relatively well-resourced and well-supported group, who were accessing government assistance with a view to expansion due to the removal of the CAP and quota restrictions for dairy farming in 2015. The majority of participants had farms of >120 acres (59.5%, *n* = 72). Three quarters of participants had completed at least secondary level education (76.0%, *n* = 92). Overall, the majority of participants were married (72.7%, *n* = 88), attended Teagasc (the Irish Agriculture and Food Development Authority) meetings at least “often” (70.2%, *n* = 85) and identified as specialist dairy type of farmer (66.9%, *n* = 81) or mixed dairy (dairy and cattle) (25.6%, *n* = 31). Most participants’ farms (95%, *n* = 115) were on a trend of expansion, having recently expanded, currently expanding or planning expansion within the next 3 years. Many participants reported incomes of over €80,000 in the past 3 years (34.7%, *n* = 42), but debt was bimodally distributed with a large proportion of participants reporting <€50,000 debt on their farms at the time of the study (42.1%, *n* = 51), and a similar proportion reporting in excess of €200,000 debt on their farms (37.2%, *n* = 45).

**Table 1:** **Demographic information of participants in the current study**.

**Age**	<35	35–44	45–54	55–64	>65	Missing
	24 (19.8%)	21 (17.4%)	41 (33.9%)	22 (18.2%)	3 (2.5%)	10 (8.3%)
**Marital status**	Single	Married	Separated	Divorced		
	25 (20.8%)	88 (73.3%)	2 (1.7%)	1 (0.8%)		4 (3.3%)
Education (ISCED 2011*)	Did not complete upper secondary school	Completed upper secondary education	Experienced tertiary level education	Completed short-cycle tertiary education	Completed bachelor level or higher	
	25 (20.7%)	29 (24%)	28 (23.1%)	10 (8.3%)	25 (20.7%)	4 (3.3%)
**Farm size (acres)**	<50	51–70	71–90	91–120	>120	
	2 (1.7%)	9 (7.4%)	9 (7.4%)	25 (20.7%)	72 (59.5%)	4 (3.3%)
**Type of farming**	Dairy mixed	Specialist dairy	Other			
	31 (25.6%)	81 (66.9%)	6 (5%)			3 (2.5%)
**Future farm direction**	Expanded farm in last 3 years	Expanding farm now	Expanding farm plan in next 3 years	Contracted farm in last 3 years or plan to in next 3 years		
	37 (30.6%)	49 (40.5%)	24 (19.8%)	6 (5%)		5 (4.1%)
**Attendance at Teagasc meetings**	Never	Rarely	Sometimes	Often	Always	
	5 (4.1%)	8 (6.6%)	19 (15.7%)	59 (48.8%)	26 (21.5%)	4 (3.3%)

*Education level is described using the levels from the International Standardized Classification of Education (2011).

The sample was recruited during attendance at Teagasc farm meetings in various locations all over the Republic of Ireland: Galway, Mayo, Roscommon, Kilkenny, and Waterford. The sample was achieved by convenience sampling through recruitment. Consequently, farmers in this group were arguably better resourced than the average dairy farmer in Ireland. Response rates for participation were fair: 300 study packs were sent out and 122 returned, which was an acceptable reliable response rate for the participants solicited [40.66%, *n* = 122; (42)]. The study was incentivized by a €100 fuel voucher that could be won by any individual who took part and returned a fuel draw letter. The individuals would be entered into a draw and the winner would be posted out the prize upon being selected. Further demographic information is displayed in Table 1.

### Measures

The current study employed a correlational design and measured six variables. Farmer’s Expectations of Injury (FEI) constituted the outcome variable and it was estimated using the “susceptibility to a farm-related accident/illness” factor of the Farm Safety and Health Beliefs Scale. Five predictors of FEI were measured: FT, FS, SS, depression, and anxiety. Based on the JDR model, FT, Farm job stress, and SS constituted measures of available resources. Depression and anxiety were included as potential mediators of the effects of these variables on Expectations of injury. FT was measured using the Financial Threat Scale (FTS), FS, using the Edinburgh Farming Stress Inventory (EFSI), and SS, using the Multidimensional Scale of Perceived Social Support (MSPSS). Depression was measured using the Patient Health Questionnaire (PHQ-8) and anxiety, using the Generalized Anxiety Disorder Assessment (GAD 7).

#### Outcome Variable – Farmers’ Expectations of Injury

In order to assess FEI, the “susceptibility to a farm-related accident/illness” factor was extracted from the Farm Safety and Health Beliefs Scale (43). The susceptibility to a farm-related accident/illness factor has established reliability (43) and reliability was confirmed in this study also (Cronbach’s α = 73). The FSHBS scale is derived from the Health Beliefs Model (44) of health and safety behaviors and includes five factors: (i) susceptibility to a farm-related accident/illness, (ii) benefits of performing safety and health behaviors; (iii) barriers to performing these behaviors; (iv) self-efficacy regarding performing these behaviors; and (v) severity/finances regarding the consequences of an accident/illness. The Susceptibility to a farm-related accident/illness factor includes six items that address the likelihood of injury on the farm (e.g., “*I’m more likely than the average farmer to have a farm-related accident or illness*”). For each statement, participants responded on a five-point scale ranging from 1 (*strongly disagree*) to 5 (*strongly agree*). There was a sixth option that represented an unknown response to the question from the participant *N/A* (*not applicable)*. Participants completed the entire FSHBS scale, which consisted of 39 items.

#### First-Order Predictors

##### Financial Threat

The FT experienced by the farmers was assessed by using the FTS (45). The FTS has an established reliability (Cronbach’s α = 0.89). The FTS was developed based on existing threat measures and threat research to assess hypothesized FT. The items include areas related to (i) risk of threat (e.g., “*How much do you feel at risk?*”); (ii) worry related to threat; (iii) anticipated threat; (iv) mental fixation on individual personal finances, and (v) uncertainty about threat. For each item, participants responded on a five-point scale ranging from 1 (*Not at all*) to 5 (*Extremely*) indicating the accuracy of statements reflecting their personal feelings about FTs they were currently facing. Participants completed the entire FTS scale, which consisted of five items.

#### Social Support

Social support was assessed by using the MSPSS (46). The MSPSS has an established reliability (Cronbach’s α = 0.88) is a brief and simple tool to use to establish the level of SS that the respondent identifies that they have. The scale was designed to assess the perceptions of SS; identified by the respondent answering questions relating to family, friends, and significant others (e.g. “*There is a special person around when I am need*”*;* “*I have friends with whom I can share my joys and sorrows*”*;* “*My family is willing to help me make decisions*”). For each question, participants responded on a seven-point scale ranging from 1 (*Very strongly disagree*) to 7 (*Very strongly agree*), indicating which statements were most representative of how much SS they felt they had, indicating their personal level of perceived SS. Participants completed the entire MSPSS scale, consisting of 12 items. Due to the small sample size, SS was included as one variable in the correlational analyses; so all items were summed to approximate a generic SS construct.

#### Farm Stress

Farm stress was assessed by using the EFSI (9). The EFSI has an established reliability (9) and reliability was confirmed in this study also (Cronbach’s α = 0.93). This inventory assess domains that are pertinent to farming lives and may be sources of added stress to the profession including (i) time pressure (e.g., “*Too much to do and too little time to do it*”, (ii) finance (e.g., “*Debt load*”), (iii) geographical isolation (e.g., “*Feeling isolated on the farm*”), (iv) hazards in farming (e.g., “*Farming related accidents*”), (v) government policy (e.g., “*Complying with environmental regulations*”), and (vi) unpredictable factors in farming (e.g., “*Bad weather*”). Answers are scored by the indicated response to a stem question “*How severe is the stress caused by this?*” For each question, participants respond on a five-point scale ranging from 1 (*None*) to 5 (*Very Severe*) indicating how each of the events and situations represented a potential source of farming-related stress and how severe the stress caused by these events/situations was when farming. Participants completed the entire adapted EFSI, consisting of 27 items. Due to the small sample size, FS was included as one variable, so all items were summed to approximate a generic FS construct.

#### Mediator – Mental Distress

In the current study, it was hypothesized that the effects of FT, SS, and FS on FEI are mediated by mental distress. To provide a measure of mental distress, we estimated the levels of depression and anxiety in our sample and used these variables to create a latent variable termed mental distress.

##### Depression

Depression levels of the farmers were assessed using The Patient Health Questionnaire (PHQ-8) (47). The PHQ-8 is a shortened form the PHQ-9, which has been employed for population studies (48). The PHQ-9 has established reliability (Cronbach’s α = 0.84) and uses a criteria-based diagnosis of depression using a shortened item scale, which can be self-administered. The questionnaire is made up of nine items and major depression is indicated if five or more of the nine symptoms have been present in previous 2 weeks. Other levels of depression are established if two, three, or four of the depressive symptoms have been present. The questions are related to specific symptoms that a person may be experiencing related to depressive feelings at any given time during the previous 2 weeks (e.g., “*Feeling down, depressed, or hopeless*” and “*Feeling bad about yourself – or that you are a failure or have let yourself or your family down*”). For each question, participants responded on a four-point scale ranging from 1 (*Not at all*) to 4 (*Nearly every day*).

In the PHQ-8, the ninth item of the PHQ-9, which refers to thoughts of suicide or self-harm, is removed. In the current study, we deployed the entire PHQ-9 scale, because we wished to include such symptoms and remove such symptoms can reduce the sensitivity of the scale at the high end (49). However, 25 participants refused to complete this item. Consequently, we employed the PHQ-8 score, which consists of eight items, with depression scores ranging from 0 to 24.

##### Anxiety

The anxiety levels of the farmers were assessed using the GAD 7 (50). The GAD 7 has an established reliability (Cronbach’s α = 0.92, 2006) and is a useful tool for identifying possible GAD with questions that inquire about the anxiety felt by the respondent in the past 2 weeks. The GAD 7 assesses the severity of the anxiety with specific questions related to worry (e.g., “*worrying about different things*”) and fear (e.g., “*feeling afraid as if something terrible might happen*”) and the inability to relax (e.g., “*trouble relaxing*”) and the effect it has had on the daily life. GAD is established if symptoms appear more often than once a week. Each answer given is accumulated to result in a score that places the respondent in the mild, moderate, or severe GAD category. For each question, participants responded on a four-point scale ranging from 1 (*Not at all*) to 4 (*Nearly every da*y). Participants completed the entire GAD 7 scale, consisting of seven items.

### Procedure

The National University Ireland, Galway Research Ethics Committee assessed and approved the study procedures with written informed consent from all subjects. All subjects gave written informed consent in accordance with the Declaration of Helsinki. The study was advertised by “word of mouth” by Teagasc dairy farming group leaders at regional centers across Ireland. Data were collected from 31st March 2015 to 31st July 2015 (4 months). All responses were verified for validity by sending study packs to the advisors directly for the dairy farmers they met with or when the researcher went to meet dairy farmers and hand out the study packs at the Teagasc meetings. Data were collected by return post with study packs handed out with a prepaid return envelope for all participants.

### Data Analysis

In the current study, we were particularly interested in relationships among our predictors and in assessing the role that mental distress plays in mediating the effects of these predictors of FEI. In other words, we hypothesized that FS and FT influence FEI and that, to some degree, this influence occurs because they cause mental distress, and SS influences FEI because it reduces mental distress. To assess these effects, structural equation modeling was employed. Structural equation modeling refers to a set of statistical methods that allow estimation of direct and indirect relationships between observed variables and latent (inferred) variables (51). In the current study, structural equation modeling was employed to estimate the effects of FS, FT, and SS on self-reported injury expectation (FEI). It was expected that these variables would affect self-reported injury expectation, but that these effects would be at least partially mediated by the effects of these variables on mental distress, a latent variable derived from measures of anxiety and depression. That is, to some degree, FS and FT increase farmers’ mental distress, but this is attenuated by SS, and increases in mental distress predict greater expectations of injury (FEI).

## Results

### Descriptive Analysis

Table 2 provides the means and SDs of the measured variables and Pearson correlations between the variables. Three variables, FT, anxiety, and depression, were log transformed to correct for skewness and kurtosis. In all three cases, values were lower than expected, suggesting that the participants had low levels of these constructs. An additional benefit of log transforming the variables was that it made the measures more sensitive at the low end of the scale. Cronbach’s alpha reliabilities of the predictor and mediator variables were above 0.8 in all cases (FT = 0.89, SS = 0.91, FS = 0.93, anxiety = 0.87, depression = 0.87) and Cronbach’s alpha for FEI was.722.

**Table 2:** **Means and SDs of the measured variables (leftmost two columns) and Pearson correlations (*r*) between the observed variables (remaining columns)**.

Scale	Mean	SD	FT (L)	SS	FS	Anx (L)	Dep (L)
Financial threat (FT)	11.17	4.11		−0.247	0.384**	0.477***	0.438***
Social support (SS)	66.55	11.29			−0.354**	−0.4**	−0.347*
Farm stress (FS)	66.68	17.76				0.463***	0.452***
Anxiety (Anx)	2.59	3.4					0.667***
Depression (Dep)	2.62	3.43					
**Farm safety and health beliefs**
Susceptibility (FEI)	2.66	0.68	0.273	−0.075	0.384**	0.339*	0.285
Benefits	4.15	0.46	−0.181	0.261	−0.029	−0.159	−0.168
Barriers	2.86	0.74	0.307*	−0.288	0.5***	0.416**	0.439***
Self-efficacy	3.5	0.54	−0.319*	0.297	−0.445***	−0.364**	−0.285
Financial effects	3.29	0.78	0.232	−0.213	0.195	0.198	0.19

Farmers’ Expectations of Injury (FEI) were measured using the Susceptibility subscale of the Farm Safety and Health Beliefs scale (see Outcome Variable – Farmers’ Expectations of Injury in section “Materials and Methods” for details). Variables followed by (L) were log transformed for analysis. Raw means and SDs are provided for these variables to facilitate interpretation. Asterisks denote significant effects (*p < 0.05, **p < 0.01, ***p < 0.001).

In order to contextualize the relationships observed among the measured variables in the current study, details of the central tendency and other features of the distributions on these variables will be briefly discussed. FEI were measured using the Susceptibility scale of the Farm Safety and Health Beliefs scale. The scores obtained constituted means of the responses to six items on a likert scale from 1 (strongly disagree) to 5 (strongly agree). These items stated a vulnerability to a farm accident and so values above 3 indicated more agreement than disagreement with such statements and indexed farmers’ perceived susceptibility to farm safety issues. The average score for all farmers was 2.66 (SD = 0.68) and 92 (77%) of farmers scored at 3 (neutral) or less. Even though this indicates that, on average, farmers did not feel vulnerable on their farms, 23% of farmers, reported values that indicated perceived vulnerability. In previous work, Hodne et al. (43) obtained a mean of 2.39 (SD = 0.54) for their sample, which was significantly less [*t* (191.17) = 4.1098, *p* < 0.001] than the average observed in the current study.

**Figure 1:**
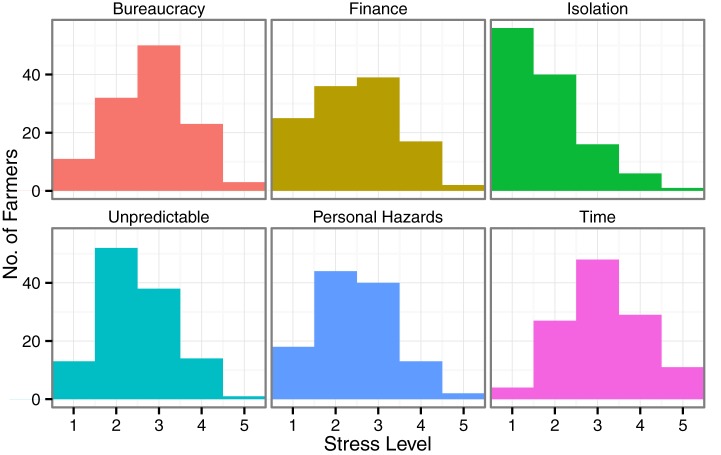
**Histogram of the values of stress level reported for each of the six areas of stress captured in the Edinburgh Farm Stress Survey**. In each case, a score of 1 indicated “no stress” and a score of 5 indicated “very severe stress.” The height of each bar denotes the number of respondents who expressed that level of stress in that stress area.

Financial threat ranged from 5 (the minimum possible) to 25 (maximum possible), and had an item level mean of 2.234, which was below the mid point of 3. This item level mean compared favorably with values obtained by Marjanovic et al. (45) (item mean: 2.74), suggesting that most farmers in the current study experienced lower FT than the standardization sample. However, three farmers reported FT scores of 20 or more indicating very high levels of financial worries. Mean FS was 66.68, which was close to the mean value of 68 reported by Deary et al. (9). The histogram in Figure 1 summarizes data from the Edinburgh Farm Stress Survey (EFSS). The highest mean stress was reported due to Time pressure (3.0), followed by Bureaucracy (2.8). Slightly lower mean stress scores were observed in the areas of Financial worries (2.4), Unpredictability of the job (2.5), and Personal hazards (2.5). The lowest source of stress was Isolation (1.7).

Farmers in the current study scored highly in SS, with a mean of 66.55. The overall item mean score was 5.55, which is slightly less than that obtained by Zimet et al. (46) in the standardization sample (5.8). Typically, scores on the MSPSS are calculated separately from Family, Friends, and Significant Others, but in the current study, all items were summed to provide a generic measure of SS. Measured this way, SS can range from 7 to 84 and scores indicated that, on average, farmers experienced high levels of SS. Only 7 of the sample provided item means of <4, which indicated neutrality with regard to statements of SS, and 89 of the farmers had item means of 6 (strong agreement) or 7 (very strong agreement).

Scores on the mental health measures were quite low indicating low levels of mental distress. On the depression scale (PHQ-8), 93 (80%) of the farmers scored 4 or less, which constitutes minimal or no depression. This proportion is greater than the proportion in this category in the population sample (75.5%) recruited by Kroenke et al. (48). A similar proportion exhibiting minimal or no anxiety was obtained for the Anxiety scale (GAD); 94 (81%) of the farmers scored 4 or less on the GAD, which was greater than the proportion in this category in a standard population sample [70.5%; (52)]. The suitability of these instruments for the current sample is considered in the discussion.

### Demographic Variables

A series of analyses of variance were conducted to assess whether demographic variables impacted FEI. No significant effects were observed in any of these variables (Age: *F*_3,103_ = 0.73, *p* = 0.54; Marital Status: *F*_1,101_ = 1.43, *p* = 0.23; Education: *F*_3,101_ = 1.09, *p* = 0.34; Farm Size: *F*_3,110_ = 0.07, *p* = 0.97; Farm Type: *F*_1,109_ = 1.94, *p* = 0.17; Farm Expansion: *F*_2,106_ = 1.63, *p* = 0.2; Meeting Attendance: *F*_2,100_ = 0.13, *p* = 0.88).

In addition, two-way ANOVAs were conducted to assess the effects of Debt (Below 100k euros/Above 100k euros) and Annual Income (Below 60k euros/Above 60k euros) on FEI and FT. Neither Debt, *F* (1,114) = 0.633, *p* > 0.05, Income, *F* (1,114) = 0.929, *p* > 0.05, nor the interaction of these variables, *F* (1,114) = 0.528, *p* > 0.05, affected FEI. Similarly, neither Debt, *F* (1,113) = 0.024, *p* > 0.05, Income, *F* (1,113) = 1.278, *p* = 0.261, nor the interaction of these variables, *F* (1,113) = 0.972, *p* > 0.05, affected FT (log transformed) scores.

### Correlations

The correlations in Table 2 provide support for the hypothesized model of FEI. FEI was predicted by FS (*r* = 0.384, *p* < 0.01) and anxiety (*r* = 0.339, *p* < 0.05). Weaker non-significant relationships were observed between FT and FEI and between depression and FEI. There was no direct relationship between SS and FEI. The proposed mediators, anxiety and depression, were predicted by the relevant first-order predictors. Anxiety was predicted by FT, FS, and anxiety, and depression was also predicted by these three measures. There was also a strong correlation between anxiety and depression, as is commonly observed.

The five proposed predictors of FEI were included in a multiple linear regression to estimate the variance explained by a linear combination of these variables. The model was significant [*F* (5, 96) = 5.462] and accounted for 18.09% of variance in FEI. In this model, only the beta value for FS was significant (*b* = 0.012, SE = 0.004, *t* = 2.880, *p* = 0.005), which suggests a more complex structure in the relationships among the variables. The remaining beta values were as follows: FT (*b* = 0.158, SE = 0.187, *t* = 0.841, *p* = 0.402), SS (*b* = 0.010, SE = 0.007, *t* = 1.468, *p* = 0.145), anxiety (*b* = 0.161, SE = 0.107, *t* = 1.498, *p* = 0.138), depression (*b* = 0.031, SE = 0.106, *t* = 0.296, *p* = 0.768).

### Structural Equation Model

As described previously, it was expected that FT, FS, and SS would impact FEI indirectly through the effects of these predictors on mental health. Following the regression analysis described above, it was apparent that FS may have a direct effect on FEI in addition to any effects mediated by mental health. A mental distress latent variable was derived from the anxiety and depression scales. Parameter estimates and fit statistics of the proposed model are provided in Table 3.

**Table 3:** **Parameter estimates of the original structural equation model**.

	*b*	SE	*β*	95% CI	*p*
**Direct effects**
On FEI					
Mental distress	0.234	0.119	0.245	(0.00, 0.47)	0.050
Farm stress	0.010	0.004	0.267	(0.00, 0.02)	0.018
On Mental distress					
Farm stress	0.013	0.004	0.332	(0.01, 0.02)	0.001
Social support	−0.016	0.006	−0.237	(−0.03, 0.00)	0.009
Financial threat (log)	0.698	0.173	0.379	(0.36, 1.04)	<0.001
**Latent variable**
Mental distress					
Anxiety (log)	1.000		0.856		–
Depression (log)	0.891	0.121	0.785	(0.65, 1.13)	<0.001
**Indirect effects**
Farm stress > Mental distress > FEI	0.003	0.002	0.081	(0.00, 0.01)	0.089
Social support > Mental distress > FEI	−0.004	0.002	−0.058	(−0.01, 0.00)	0.110
Financial threat (log) > Mental distress > FEI	0.163	0.090	0.093	(−0.01, 0.34)	0.071

*CFI stands for comparative fit index, TLI stands for Tucker–Lewis index. RMSEA stands for root mean square error of approximation. Values obtained from lavaan (version.5-20) R package (66, 67)*.

*Note: χ^2^(5) = 3.687, *p* = 0.595; CFI = 1.0; TLI = 1.024; RMSEA = 0.000 (90% CI = 0.00–0.12)*.

In line with the proposed model, significant direct effects of mental distress and FS on FEI were observed. As mental distress and FS increased, FEI increased. There were also significant direct effects of FS, FT and SS on mental distress. As expected, mental distress was increased by FT and FS, but reduced by SS. The indirect effects of FS, FT, and SS on FEI were not significant, but were in the expected direction. SS negatively correlated with FS and FT, suggesting potential benefits of SS in reducing these sources of mental distress.

The proposed model was compared to alternative models to assess whether it provided the most appropriate model of the obtained data. First, the latent variable of mental distress was removed and anxiety replaced it as the mediator of first-order effects on FEI. The resulting model (anxiety mediation) demonstrated good fit with a non-significant chi-square test (3.106, *p* = 0.212), IFI value of.984 and a CFI value of.985. This model had an AIC value of 2555.57, which was marginally better than the AIC value of the proposed latent variable model (2628.17), but not significantly so [χ^2^(3) = 0.58171, *p* = 0.9006], suggesting the simpler model was the more parsimonious alternative. However, the fit statistics for the simpler model were not as good as the proposed model. The chi-square (3.687, *p* = 0.595), CFI (1.0), and IFI (1.01) values were all superior for the original proposed model. The RMSEA was lower for the proposed model (0.000) than the simpler model (0.072) and the 90% confidence interval was tighter (proposed model: 0.00–0.12, simpler model: 0.00–0.22).

Farm stress is a heterogeneous construct and the EFSS includes FT and isolation as components of the FS measure (see Figure 1). To assess whether some of the effect of FT or SS might have been mitigated by these subscales of the FS measure, we estimated the correlation between the Financial and Isolation subscales of the FS measure and the log-transformed FT score and the SS measure. The obtained correlation between the financial measures was *r* = 0.642, *p* < 0.0001, a strong correlation and the correlation between isolation and SS was *r* = −0.250, *p* = 0.006, a weak to moderate correlation. Given the strength of the correlation between the financial measures, we developed a non-financial FS score by excluding scores from the Financial subscale of the FS measure. We then included this non-financial FS as a first-order predictor in place of the original FS measure. Results of this analysis are presented in Table 4.

**Table 4:** **Parameter estimates of the revised structural equation model that replaced the original farm stress measure with the non-financial farm stress measure**.

	*b*	SE	*β*	95% CI	*p*
**Direct effects**
On FEI					
Mental distress	0.283	0.118	0.295	(0.05, 0.51)	0.016
Farm stress (non-financial)	0.009	0.005	0.202	(0.00, 0.02)	0.067
On Mental distress					
Farm stress (non-financial)	0.015	0.004	0.323	(0.01, 0.02)	0.001
Social support	−0.015	0.006	−0.226	(−0.03, 0.00)	0.014
Financial threat (log)	0.776	0.168	0.423	(0.45, 1.10)	<0.001
**Latent variable**
Mental distress					
Anxiety (log)	1.000		0.852		–
Depression (log)	0.898	0.121	0.787	(0.66, 1.13)	<0.001
**Indirect effects**
Farm stress > Mental distress > FEI	0.004	0.002	0.095	(0.00, 0.01)	0.048
Social support > Mental distress > FEI	−0.004	0.002	−0.067	(−0.01, 0.00)	0.078
Financial threat (log) > Mental distress > FEI	0.219	0.099	0.125	(0.03, 0.41)	0.027

*CFI stands for comparative fit index, TLI stands for Tucker–Lewis index. RMSEA stands for root mean square error of approximation*.

*Note: χ^2^(5) = 4.257, *p* = 0.513; CFI = 1.0; TLI = 1.014; RMSEA = 0.000 [90% CI = 0.00–0.13]*.

**Figure 2:**
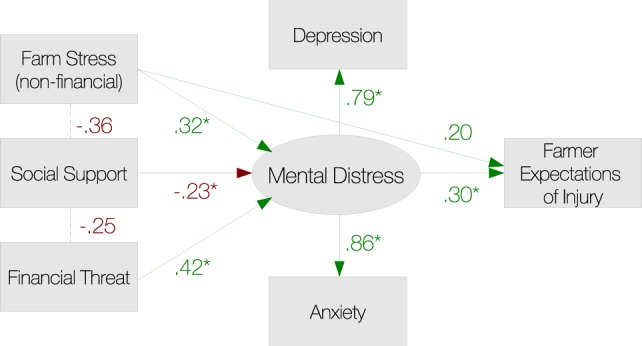
**Path diagram of the model of farmers’ expectations of injury supported by the current analyses**. Asterisks denote relationships that are significant at *p* < 0.05.

Most of the effects in the revised model were the same as those observed in the proposed model. There were a number of differences however. The non-financial FS measure was not a significant direct predictor FEI, but mental distress was. In addition, the indirect effects of non-financial FS and FT were both significant in the revised model. Nevertheless, even though the significance of some effects was affected in the new model, the patterns of correlation were largely similar. Since the revised model more clearly estimated the effects of FT, it was preferred to the original proposed model (see Figure 2).

To test the deleted paths in the revised model, paths from SS and FT to FEI were added to the proposed model (full model). This model had an AIC value of 2243.1, which was marginally worse than the AIC value of the proposed model (2242.6) but not significantly so [χ^2^(2) = 3.5045, *p* = 0.1734]. The direct paths from SS and FT to FEI were not significant in the full model, suggesting that their deletion from the proposed model was appropriate. Two trimmed models were compared to the proposed model. In the first reduced model (reduced model 1), the direct path from FS to FEI was removed. The second reduced model had an AIC value of 2243.8, which almost identical, but slightly worse than the AIC value of the proposed model [χ^2^(1) = 3.2439, *p* = 0.07]. All of the fit statistics (e.g., RMSEA, CFI) for this reduced model were worse than the revised model. In the second reduced model (reduced model 2), the direct path from mental distress to FEI was removed. The second reduced model had an AIC value of 2244.5, which was also very similar, but slightly worse than the AIC value of the proposed model [χ^2^(0) = 1.9223, *p* = 0.02135]. Once again, all of the fit statistics (e.g., RMSEA, CFI) for this reduced model were inferior to those of the revised model.

## Discussion

The current study investigated relationships between FT, SS, farm job stress, mental distress, and FEI. The findings support the conclusion that mental distress mediates the effects of non-financial FS and FT FEI. Indirect influences of SS on FEI were in the expected direction but non-significant. That is, non-financial FS and FT contribute to mental distress and mental distress affects FEI. Non-financial FS and FT significantly increased mental distress and SS significantly reduced mental distress. Significant indirect relationships were found between FEI and FT and farm job stress. Non-financial FS did not significantly predict FEI directly, but the path improved the fit of the model, suggesting that stress may have direct effects on FEI. This suggests that some of the effects of stress are not mediated by mental distress.

One interpretation of the patterns of relationships observed in the current study is that general FS and financial worries reduce the farmer’s available resources to deal with the farm and that SS supplements those resources. Such an interpretation is in line with the Conservation of Resources theory described earlier. Interventions for farmers would, thus, be best directed at reducing known stressors and financial uncertainty for farmers and ensuring that farmers are receiving SS. The trend toward mechanization and infrastructural investment in farming means that farmers spend more time lone working, which increases stress and reduces SS. In addition, the transmission of safety best practices through the farming population is likely best facilitated through peer networks of farmers speaking to farmer (53). Consequently, initiatives that seek to connect farmers can have multiple benefits for farm safety and productivity.

Previous research has indicated that farmers suffering from mental distress are less likely to engage with health and safety leading to injury (54). The current findings suggest that farmers are, to some degree, aware of the compromises that they feel they need to make for the farm to survive. The CoR approach suggests that it is possible for farmers to become embroiled in a negative spiral in which dysfunctional coping strategies, such as “cutting corners,” lead to accidents that further reduce the farmer’s ability to run the farm. Given that farmers are required to make substantial financial investments to run their farms, such negative spirals, can result in farmers losing their farms and their livelihoods.

Improving the health and safety of farmers is necessary for the viability of the profession, has implications for national food security, and, in Ireland, is essential to the export economy. Consequently, there is an obligation on policy makers to facilitate enhancements in this area (55). Farmers contact a range of stresses and, in many cases, current business models often expose them to high levels of financial risk. Traditional sources of SS have also been somewhat eroded in recent years. How best to develop policy interventions to support farmers has been an important goal of the field of agricultural extension. An important concept in this literature is the agricultural knowledge and information system (AKIS), in which farmer is centrally positioned with access to multiple sources of knowledge and information from research, extension, and education (56). The AKIS approach may provide a framework through which to develop interventions to enhance farmer health and safety. With a particular focus on mental health, greater collaboration between farmer representative groups, development groups, and government departments of health and agriculture will facilitate more appropriate intervention. Farmers would also benefit from interventions, such as mental health first aid (57, 58), that normalize healthy coping strategies (59) and minimize exposure to mental health stigma.

The current data provide an interesting snapshot of the Irish dairy farmer in 2015. The majority of farmers did not feel susceptible to injury, but the average for the sample was significantly higher than that found by Hodne et al. (43). There are a number of differences between the samples. First, the farmers in the current sample were exclusively dairy farmers, whereas the sample recruited by Hodne et al. was mixed (33% produced cattle, 41% hogs). In Ireland, dairy farming contributes a higher proportion of fatalities than other types of farming, so this may explain the difference in expectations of injury. The average FT experienced by the sample was lower than that recorded by Marjanovic et al. (45), suggesting that the sample felt relatively financially secure. Consequently, in the analyses we conducted, this variable was log transformed to correct for skew and to enhance the sensitivity of the scale at the low end. In addition, the vast majority of the sample had either recently expanded or were about to expand in the coming years, so there might have been a selection effect that prioritized farmers who were on a better financial footing. However, the stress levels observed in the population were very similar to those reported by Deary et al. (9). Nevertheless, such selection effects would likely have reduced the obtained correlations and the strength of associations found among the variables. Part of the challenge of measuring the effects of stress on farmers is that those farmers who are most stressed are least likely to be willing to spend time completing surveys. Though we had considerable buy-in from the farming community, it is still difficult to access those most in need of financial and statement concerns both financial support and social support.

We employed two measures of mental distress that are designed to identify clinical levels of anxiety and depression. Both these scales have previously been employed with population samples (48, 52) and, given the stress that farmers contact at work, we expected to observe greater levels of mental distress in the sample that were obtained. There are three possible reasons for this effect. First, the aforementioned selection effect might have meant that we included dairy farmers who were less stressed and more financially secure. Such farmers were more likely to come into contact with the researchers and more likely to complete surveys. Second, farmers might be more resilient (60) to stress and financial worry than the general population. Finally, it is possible that farmers may be unwilling to acknowledge symptoms of poor mental health due to a “macho” (59) perception that such symptoms constitute evidence of weakness. It is not possible to adjudicate between these possibilities with the data available to us. As with FT, if a greater spread of mental distress was obtained, it would provide clearer relationships with injury expectations. As with FT, log transformations were employed that corrected for skew and increased the sensitivity of the scales at low end. For future research, however, we would recommend employing a scale that may be more sensitive to lower levels of depression in the general population, such as the Centre for Epidemiologic Studies Depression scale [CES-D; (61)], as an alternative.

The impact of FT on farmers’ mental wellbeing constitutes a microcosm of the effects of FT on mental health in the rest of the population. The recent recession had considerable effects on population mental health through greater FT, especially through unemployment (62, 63). For many of us, we feel a moral imperative that financial conditions that are largely beyond an individual’s control should not cause excessive suffering. However, the case for protecting citizens from FT is not just moral, it is also economic. When individuals suffer mental distress, they are less productive at work and may need to abstain from work resulting lost productivity. Treating mental health problems is also very costly. In the USA in 2000, the costs (indirect and direct) of depression alone have been estimated at $76 billion (64, 65). It is clear that, in the case of FT, prevention of mental health problems is better than cure from both an economic and social justice perspective.

## Author Contributions

DO, EF, and JM designed the research study. EF and JM conducted the research. EF, DO, and JM wrote the paper and all authors proposed revisions. CN provided statistical analysis expertise.

## Conflict of Interest Statement

The authors declare that the research was conducted in the absence of any commercial or financial relationships that could be construed as a potential conflict of interest.
